# Right to science principles should guide global governance on health

**DOI:** 10.3389/fsoc.2023.1271063

**Published:** 2023-11-21

**Authors:** Gisa Dang, Mike Frick

**Affiliations:** Tuberculosis Project, Treatment Action Group, New York, NY, United States

**Keywords:** right to science, pandemic treaty, tuberculosis, universal health coverage, global health

## 1 Introduction

If the distribution of COVID-19 vaccines had been more equitable across the world, it would have prevented three million infections and 1.3 million people who lost their lives would still be alive (Moore et al., 2022).^1^ This stark finding should convince political leaders that inequitable access to scientific benefits in the face of global health crises violates human rights. COVID-19 will not be the last pandemic humanity faces, and even today is not the only epidemic that requires governments to see scientific progress as a fundamental human right.

In April 2023, the World Health Organization (WHO) declared an end to COVID-19 as a global public health emergency but stressed that the difficult work of recovering from COVID-19 and preparing for future pandemics is just beginning (World Health Organization, 2023). This work—termed “pandemic prevention, preparedness, and response” (PPPR)—is unfolding through a record number of international negotiations on global health issues. In September 2023, the United Nations General Assembly (UNGA) held three high-level meetings on health: one on tuberculosis (TB), one on universal health coverage (UHC), and one on PPPR. Concurrently, WHO member states are negotiating a legally binding Pandemic Accord and revising the International Health Regulations. For the first time in these fora, stakeholders are invoking the right of everyone to enjoy the benefits of scientific progress and its applications (hereafter: right to science) by name and appealing to the right's principles to frame the commitments governments will make to safeguard health.

This viewpoint analyzes how the right to science is being interpreted by governments and civil society in these political processes. Although often discussed in relation to the right to health, we argue that the right to science should stand as a distinct guidepost for human rights-based governance of global health challenges, particularly with respect to international cooperation, access to health technologies and information, and participation in science and decision-making.^2^

### 1.1 Right to science in UN high-level meeting multi-stakeholder hearings

In May 2023, the UN held a series of “multi-stakeholder hearings” to prepare for the three high-level meetings on health. These hearings gave civil society an opportunity to share their recommendations with member states with the goal of influencing the political declaration to be adopted at each meeting during UNGA.^3^ Our analysis of oral and written statements delivered at these hearings shows distinct differences in how participants addressed human rights overall, and the right to science specifically.

The UHC meeting centered human rights from the start and throughout panels and verbal interventions. Without directly mentioning the right to science, speakers invoked the right's concepts of participation in policymaking, investment in health technology development, access to innovations (defined by the AAAQ standard: Availability, Accessibility, Acceptability, and Quality), and government accountability.^4^

Few statements at the PPPR hearing referenced human rights. Several speakers pointed to a lack of international solidarity; refusal to share technologies and resulting inequitable access to vaccines; and non-inclusion of civil society in decision making. Advocates requested the political declaration recognize inequities caused by intellectual property barriers and asked governments to place conditionalities on public financing for R&D to enable access and technology sharing. Except for one statement that PPPR is a human rights issue, and one broad reference to economic, social and cultural rights, government shortcomings in pandemic response were not framed in human rights terms, suggesting that human rights are not yet understood as an inherent component of PPPR (President of the General Assembly, 2023a).

The TB meeting was the only one in which participants directly referenced the right to science (Treatment Action Group, 2023). Speakers also framed issues in right to science language. Floor interventions pointed to the right of groups vulnerable to TB to benefit from scientific progress; asked states for quicker uptake of new WHO treatment guidelines under the obligation to share the latest medical advancements; and referenced the need for making decisions based on scientific evidence (President of the General Assembly, 2023b).

### 1.2 Right to science in UN high-level meeting outcome documents

Early in negotiations on the TB High-Level Meeting political declaration member states proposed adding explicit references to the right to science. The first mention was inserted by two different member states in the preambular section of the declaration; during negotiations this became:

“Reaffirm the Universal Declaration of Human Rights, the International Covenant on Economic, Social and Cultural Rights, the right of everyone to the enjoyment of highest attainable standard of physical and mental health, and that the fulfillment of the right to health in the context of tuberculosis is closely linked to the right to enjoy and share the benefits of scientific progress and its applications […],”^5^

The language goes on to center “access to the benefits of research and innovation” as a particular concern. Member states also introduced the right to science in paragraph 39, the first in the declaration to begin with the word “commit”:

“Commit to protect and promote [the right to health], and the right to enjoy the benefits of scientific progress and its application in order to advance towards universal access to quality, affordable, inclusive, equitable and timely prevention, diagnosis, treatment, care, and awareness raising related to tuberculosis, and address its economic and social determinants.”^6^

As in the preambular paragraph, the right to science follows a reference to the right to health and is justified in relation to access to TB services and tools. At no point in the negotiations did any member state propose major substantive edits to these sections, signaling a general consensus.

Other areas of the TB declaration allude to language found in the UDHR (Art. 27) and ICESCR (Art. 15) establishing the right. One paragraph expresses states' commitment “to create a research-enabling environment that […] promotes collaboration in TB research and development (R&D) across UN Member States in order to develop and introduce new tools […] and to ensure equitable access to the benefits and applications of TB research.” Another paragraph refers to “the importance of global collaboration and increased investment to fast-track progress and ensure equitable access and maximal return on public investment in scientific progress.” This idea is echoed in a later paragraph: “Commit to maximize the potential of innovation to end tuberculosis by 2030, including through international cooperation as well as financing, encouraging greater collaboration between the scientific research and innovation community and TB stakeholders […].” Together, these parts of the declaration address all three state obligations to develop, diffuse, and conserve science and its benefits articulated in ICESCR Art 15.^7^

The directness and breadth with which the TB declaration discusses the right to science stands apart from the UHC and PPPR political declarations, which open by reaffirming the right to health but do not connect it to the right to science—an outcome foreshadowed by the relative lack of attention to the right at the UHC and PPPR multi-stakeholder hearings. The UHC outcome document does not reference the right to science despite addressing innovation in several sections, and the PPPR declaration only mentions the right in a paragraph on digital health.

### 1.3 Right to science in the pandemic instrument

At the time of writing, the International Negotiating Body (INB) had just concluded its sixth meeting (17-21 July 2023) to discuss the Bureau's text of the WHO convention, agreement or other international instrument on pandemic prevention, preparedness and response (CA+) (WHO, 2023), a precursor to the official first draft of the Pandemic Accord (WHO, 2023). It is unclear what provisions will enter the first draft and survive negotiations, but the relevance of the right to science for PPPR is underlined throughout this starting document.

The WHO CA+ covers access to tangible and intangible applications of science. Regarding tangible outputs, the CA+ discusses development of pandemic products (Article 9), access and benefit sharing (Article 12), and manufacturing and supply (Article 13) (Health Policy Watch, 2023). Concerning intangible outputs, the draft speaks of making policy on the “best available science and evidence;” “open science approaches;” and strengthening “science, public health and pandemic literacy in the population;” and promotion of international cooperation.

Right to science concepts are present in the CA+ but rendered in altered form. Where the ICESCR frames access under the right to science through distinct state responsibilities to *develop, diffuse and conserve* scientific advances, the CA+ subsumes these actions under the idea of *equity*: “equity includes the unhindered, fair, equitable and timely access to safe, effective, quality and affordable pandemic-related products and services, information, pandemic-related technologies and social support.” Similarly, where the right to science speaks of *international cooperation*, this appears in the CA+ under the term *solidarity*: “[e]ffective national, international, multilateral, bilateral and multisectoral collaboration, coordination and cooperation to achieve the common interest of a safer, fairer, more equitable and better prepared world […].” The concepts of *solidarity* (CA+) and *international cooperation* (ICESCR) as well as *equity* (CA+) and *development, diffusion, and conservation* (ICESCR) appear overlapping—but these areas of the CA+ would be stronger if grounded in right to science obligations established in human rights law. The right to science does garner one direct mention in the conceptual zero draft (February 2023) in a preambular paragraph (not yet included in CA+) on the right to health and intellectual property (WHO, 2022).

## 2 Discussion: from paper to practice

Not knowing the outcomes of these political processes, advocates and governments are already working on initiatives to address the failings of the COVID-19 response. Two efforts stand out for the potential—yet unrealized—to enact “alternatives” to the status quo driven by strong right to science principles on international cooperation, access, and participation.

### 2.1 International cooperation and access at the mRNA hub

WHO Director-General Dr. Tedros has stated that “a Pandemic Accord that fails to ensure equitable access to pandemic-related products fails. We do not have to choose between equitable access and innovation (Quoted in Geneva Health Files, 2023).” In pursuit of this aspiration, the mRNA Vaccine Technology Transfer Hub (the Hub) was founded in 2021 to democratize the ability of countries to develop and manufacture mRNA vaccines against COVID-19 and other diseases (e.g., TB and HIV). Supported by WHO and the Medicines Patent Pool, the Hub consists of three South African partners: Afrigen Biologics, Biovac, and the South African Medical Research Council. Vaccines and associated technologies developed at the Hub will be shared with designated vaccine manufacturers (“spokes”) in 15 countries (WHO, 2021). Any technology improvements made by the spokes will be shared back with the Hub for distribution to other partners. By innovating through sharing, the mRNA Hub aspires to an “end-to-end transformation of PP[P]R starting at the beginning: with research and development geared toward technology transfer and access (Torreele et al., 2023).” In right to science parlance, the Hub has created a platform for governments to address development, diffusion, and conservation of scientific progress simultaneously as mutually reinforcing responsibilities.

### 2.2 Participation and PPPR initiatives

The right of affected communities to participate in science and policymaking has been a hard-won legacy of the AIDS movement (Dang and Frick, 2021). While advocates in other diseases areas (e.g., TB) have won some recognition of the same participation principle, states' reactions to PPPR continue to put up roadblocks to the participation tenet of the right to science.^8^ Examples include the initial non-inclusion of civil society representatives in the World Bank FIF, a newly established multilateral pandemic funding mechanism (Kates et al., 2023). The Pandemic Accord has been criticized for not providing space for civil society participation (The Independent Panel, 2022), and recently, a group of 62 member states called for a more inclusive process that would include civil society in closed-door discussions and institute channels for state/civil society interactions (Geneva Health Files, 2023). A WHO project to design a medical countermeasures platform (MCP) to develop and secure equitable access to pandemic tools made a gesture to incorporate civil society (Rahman et al., 2023), yet civil society representatives have been excluded from meetings (Baker et al., 2023). Significant time could be saved, and insight gained, if participation in science and health policy making was institutionalized as a norm in line with right to science obligations.

## 3 Conclusion

Once called the “forgotten human right,” (Chapman, 2009) the right to science is starting to be recognized by name in political negotiations on health, and its foundational concepts and principles occupy a more prominent position than before. Initiatives on TB, UHC, and PPPR could become vehicles for states to fulfill their right to science obligations. Centering the right to science in these high-level political fora and in the Pandemic Accord will help establish a practical normative benchmark for guiding states as they confront global health challenges in ways that increase international cooperation, foster participation, and ensure equitable access to the fruits of scientific advancement. State and non-state actors should build on this nascent political recognition of the right to science to strengthen effective human rights-based global health governance.

## Author contributions

GD: Writing—original draft, Writing—review & editing. MF: Writing—original draft, Writing—review & editing.

## Footnotes

^1^Modeling for end of 2021.

^2^The Right to Science is prescribed in the Universal Declaration of Human Rights (UDHR) Article 27 and the International Covenant on Economic, Social, and Cultural Rights (ICESCR) Articles 15 (1) (b), (2), (3) and (4). The right recently received an authoritative analysis by the Committee on Economic, Social and Cultural Rights in General Comment 25 (GC). The right to science requires states as duty bearers to develop, diffuse, and conserve scientific progress and its benefits. These duties extend to both tangible and intangible results of science as well as the scientific process itself. Tangible results include, for example, vaccines or diagnostic platforms; intangible results include access to knowledge and information and the means, methods, and materials of scientific discovery. Central to the right are concepts such as international cooperation and broad participation in science.

^3^UN High-Level Meetings Multi-Stakeholder Hearings are an official UN process in which stakeholders other than member states can share their analyses and recommendations regarding a certain topic. As an official instrument, these hearings require an application process, seats are limited, and speaker times allotted as per UN rules. These hearings are an important avenue for civil society to share their priorities with and ask questions of member states in the lead up to the High-Level Meetings during UNGA that will pass an official political declaration on each topic.

^4^For documentation on the UHC HLM Multi-Stakeholder Meeting see e.g., uhc2030 (2023a) and Uhc2030 (2023b).

^5^United Nations (2023). Paragraph 5 in official text adopted 5 October 2023.

^6^United Nations (2023). Paragraph 39 in official text adopted 5 October 2023.

^7^United Nations (2023). Paragraphs 69, 32, and 67 in official text adopted 5 October 2023.

^8^CESCR GC25 highlighted participation in its formulation of the right to science as the right to benefit from and participate in scientific progress. The right to participation is, of course, a human right on its own as well as an important part of the right to health.
